# Real-time spectroscopic monitoring of photocatalytic activity promoted by graphene in a microfluidic reactor

**DOI:** 10.1038/srep28803

**Published:** 2016-06-27

**Authors:** Yifan Li, Beichen Lin, Likai Ge, Hongchen Guo, Xinyi Chen, Miao Lu

**Affiliations:** 1 Pen-Tung Sah Research Institute of Micro-Nano Science & Technology, Xiamen University, Xiamen 361005, China

## Abstract

Photocatalytic microreactors have been utilized as rapid, versatile platforms for the characterization of photocatalysts. In this work, a photocatalytic microreactor integrated with absorption spectroscopy was proposed for the real-time monitoring of photocatalytic activity using different catalysts. The validity of this method was investigated by the rapid screening on the photocatalytic performance of a titanium oxide (TiO_2_)-decorated graphene oxide (GO) sheet for the degradation of methylene blue under monochromatic visible irradiation. The sampling interval time could be minimized to 10 s for achieving real-time detection. The best photocatalytic activity was observed for an optimized TiO_2_/GO weight mixing ratio of 7:11, with a reaction rate constant up to 0.067 min^−1^. The addition of GO into TiO_2_ enhances photocatalytic activity and adsorption of MB molecules. The synthetic reaction rate constant was up to approximately 0.11 min^−1^, which was also the highest among the catalysts. The microreactor exhibited good sensitivity and reproducibility without weakening the performance of the photocatalysts. Consequently, the photocatalytic microreactor is promising as a simple, portable, and rapid screening tool for new photocatalysts.

Photocatalysis is an important approach for environmental purification as it can decompose a wide range of organic pollutants into innocuous products under irradiation by sunlight^1^,^2^. In addition, it has attracted extensive attention for the harvesting of solar energy, caused by its potential capacity for converting photon energy into chemical energy^3^,^4^. Various photocatalytic microreactors have been developed for the purpose of obtaining a large surface-to-volume ratio, short diffusion distance, and uniform irradiation^5^,^6^,^7^. Particularly, they are useful tools for the rapid screening of photocatalysts, with significantly reduced consumption of catalysts and reagents^8^,^9^. However, the reagent in these microreactors has to be collected from the outlet, followed by further characterization by certain bench-top analytical instruments^10^,^11^, which is not only time-consuming but also hinders the real-time monitoring of the photocatalytic activity.

Meanwhile, the combination of microfluidic chemical or biological reactors with *in situ* spectroscopic monitoring, such as absorption spectroscopy^12^,^13^,^14^,^15^,^16^,^17^,^18^, infrared spectroscopy^19^,^20^, surface-enhanced Raman spectroscopy^21^,^22^,^23^, and laser-induced fluorescence spectroscopy^24^,^25^, have been reported thus far as these techniques involve merits such as real-time analysis as well as the enhancement of light–matter interaction for improving the limit of detection and the use of compact cellular analysis modules for replacing the traditional bulky, expensive analytical instruments.

Herein, we proposed a real-time microphotocatalytic reactor equipped with absorption spectroscopy for investigating photocatalytic activities. Although some microfluidic-chip-based systems have been reported for the rapid screening of photocatalysts^26^, which employed a CCD (Charge-coupled Device) camera to obtain the real-time gray value of the solution under light irradiation, they could barely determine the precise concentration of the reactants. In contrast, the proposed microreactor uses a spectrometer to obtain absorption spectra, and the performance of photocatalysis, i.e., the relationship between optical spectra and the change in the solution concentration, can be determined by the Beer–Lambert law^27^,^28^,^29^.

As a proof-of-concept, the photocatalytic performance of a titanium dioxide (TiO_2_)-decorated graphene oxide (GO) sheet with different weight mixing ratios was evaluated using the proposed microreactor for the degradation of methylene blue (MB) under visible-light irradiation. Because of the extremely large specific surface area, high thermal and chemical stabilities, and excellent mobility of charge carriers, GO has become one of the most ideal supports for the separation and transfer of the photogenerated charge carriers^30^,^31^,^32^. Thus, various GO-based photocatalysts, such as TiO_2_/GO^33^,^34^,^35^,^36^, ZnO/GO^37^, and Ag nanocompound/GO^38^,^39^,^40^, have been developed for the photocatalytic degradation of organic pollutants. However, the mechanism of how GO promotes photocatalytic performance is not completely clear thus far, and superior photocatalytic activity is still highly required for applications.

In this paper, we proposed a micro-photocatalytic reactor integrated with monochromatic measuring system to achieve real-time spectroscopic monitoring. This microreactor demonstrates potential for becoming a simple, portable, and rapid screening tool for new photocatalysts. The proposed microreactor simplifies the characterization of photocatalysis by saving the complicated continuous light sources, replaced with swappable monochromatic sources. It can be also useful for time sensitive investigation of the photocatalytic mechanism, with a minimized interval at approximately 10 s.

## Results and Discussion

**Figure 1:**
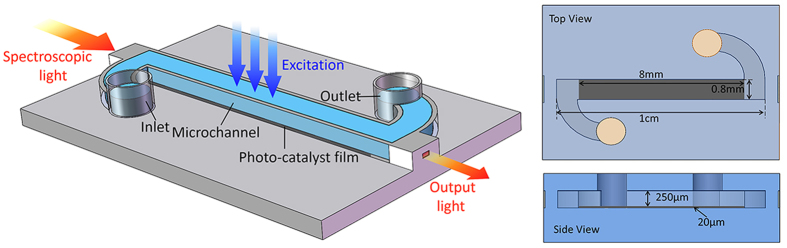
Schematic diagram of the fabricated microreactor.

Figure 1 shows the architecture of the microreactor. The photocatalyst membrane (~20 μm) was integrated into a transparent microchannel with specified size and shape, as described in the top view and side view of Fig. 1. Two ports were drilled at the two ends of the microchannel as the inlet and outlets ports, from which the MB solution could be injected into and pumped out, respectively. The inlet and outlet ports should be settled at the side of the straight part of the microchannel, to ensure that the spectroscopic light (for measurement) could go through the straight area of the microchannel without potential interference induced by the ports. The photocatalytic light was place above the microreactor. Both the spectroscopic and photocatalytic light sources were monochromatic light emitting diodes (LEDs), so that the light sources could be easily swapped based on the circumstance for portable applications. In addition, TiO_2_/GO as a mature and effective photocatalyst, was employed to characterize the performance of the microreactor.

### Photocatalytic activity of TiO_2_-decorated GO sheets

**Figure 2:**
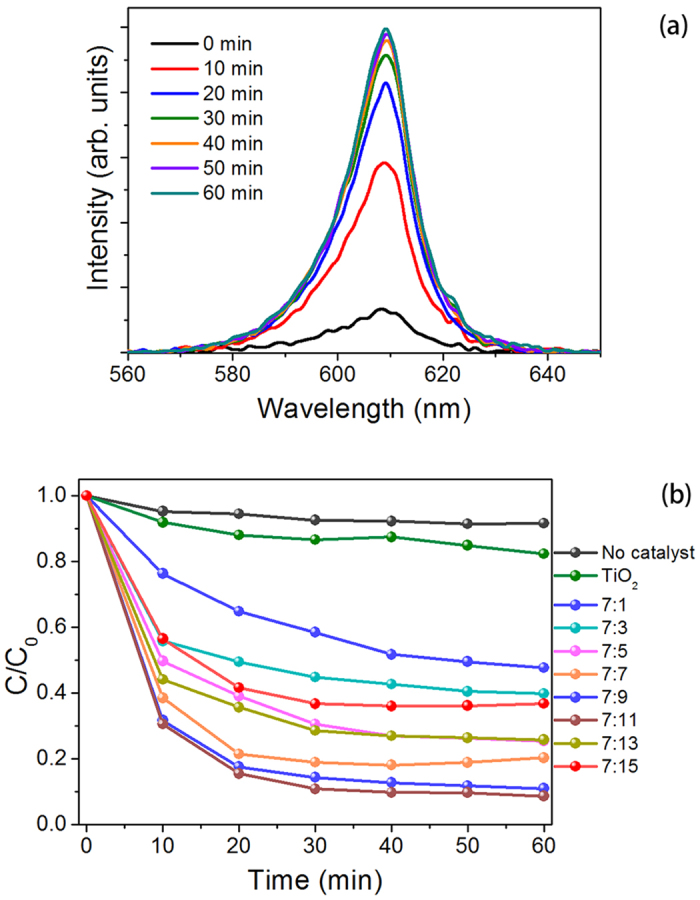
(**a**) Absorption spectra during the photodegradation of MB. (**b**) normalized MB concentration during photocatalytic degradation.

Figure 2(a) shows the *in-situ* measured spectra during the photocatalysis under spectroscopic light source of 609 nm i.e. the increase of the measured light intensity implied the degradation of the MB solution. The comparison of the degradation of various TiO_2_/GO composites under the same spectroscopic light source was summarized in Fig. 2(b). No significant change was observed in the device without the embedded photocatalysts sheet, which served as the control. In general, the TiO_2_-decorated GO sheet exhibited better performance for the photocatalysis of MB, with the optimum ratio of TiO_2_/GO being approximately 7:11. It should be noticed from Fig. 2(b) that the degradation went fast in the first 10 min.

Particularly for the first 10 min, the real-time monitoring of the photocatalytic degradation of MB was conducted by recording the absorption spectrum in each 10 s. The reaction rate constants can be deduced from the change of MB concentration i.e. absorbance by the following relationship^11^:


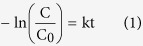


Here ln represents the logarithm of natural base, t represents the effective reaction time, and C_0_ and C represent the initial concentration and the concentration after degradation, respectively. k represents the reaction rate constant, which is normally chosen as the basic kinetic parameter for the comparison of different photocatalysts.

**Figure 3:**
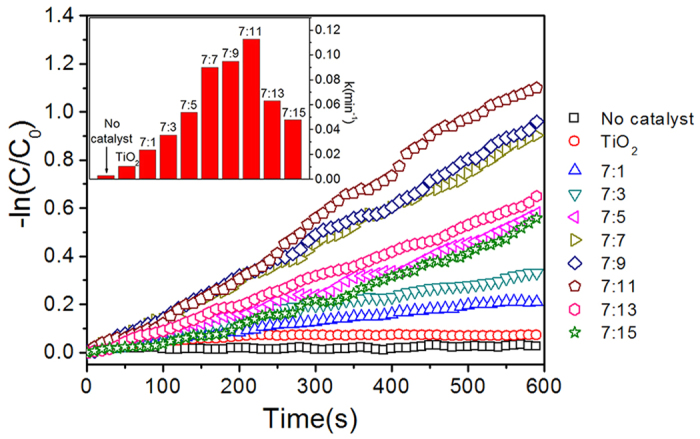
Degradation of MB as a function of the photocatalytic time. The inset shows the reaction rate constants with different photocatalysts.

Figure 3 summarized the reaction rate constant of various photacatalysts. k_TiO2_ was approximately 0.01 min^−1^; in comparison, k_TiO2/GO=7:11_ was up to 0.11 min^−1^, which is approximately one order of magnitude higher. Obviously, in term of rate constants, TiO_2_-decorated GO sheets surpassed bare TiO_2_, which further confirmed previously published studies^41^,^42^,^43^. Moreover, the rate constants of TiO_2_/GO integrated in our microreactors were comparable to these studies, which proved that the configuration of our system (both the microreactor and the measurement) did not weaken the performance of the photocatalysts.

### Analysis of the microreactor

Photocatalysis of bare TiO_2_ might be restricted by broad band gap and low adsorption capacity of TiO_2_. It has been reported that TiO_2_-decorated GO sheets could increase the photocatalytic performance compared to bare TiO_2_ in two ways^44^.

**Figure 4:**
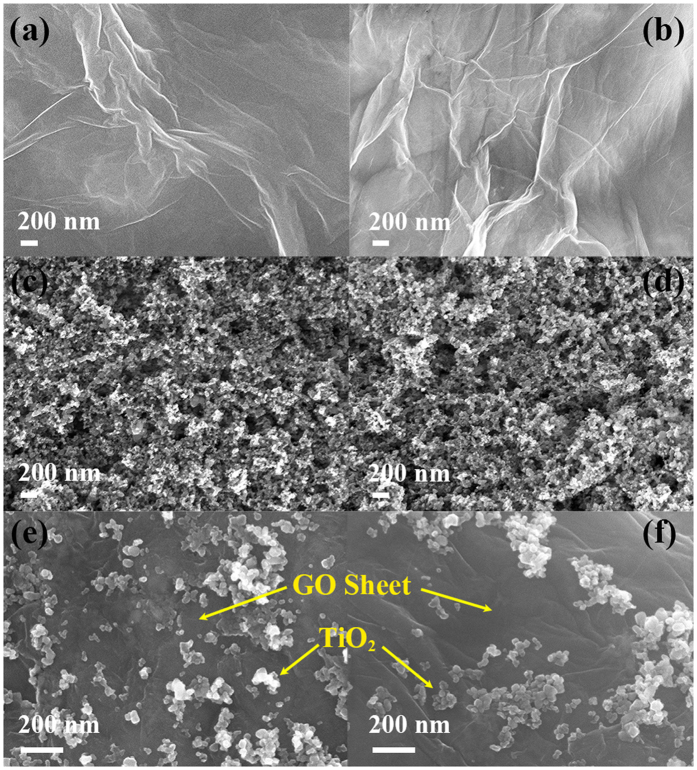
SEM images of the photocatalysts (**a,b**) GO; (**c,d**) TiO_2_; (**e,f**) TiO_2_/GO at a weight mixing ratio of TiO_2_:GO = 7:11, before (left column) and after (right column) reaction.

**Figure 5:**
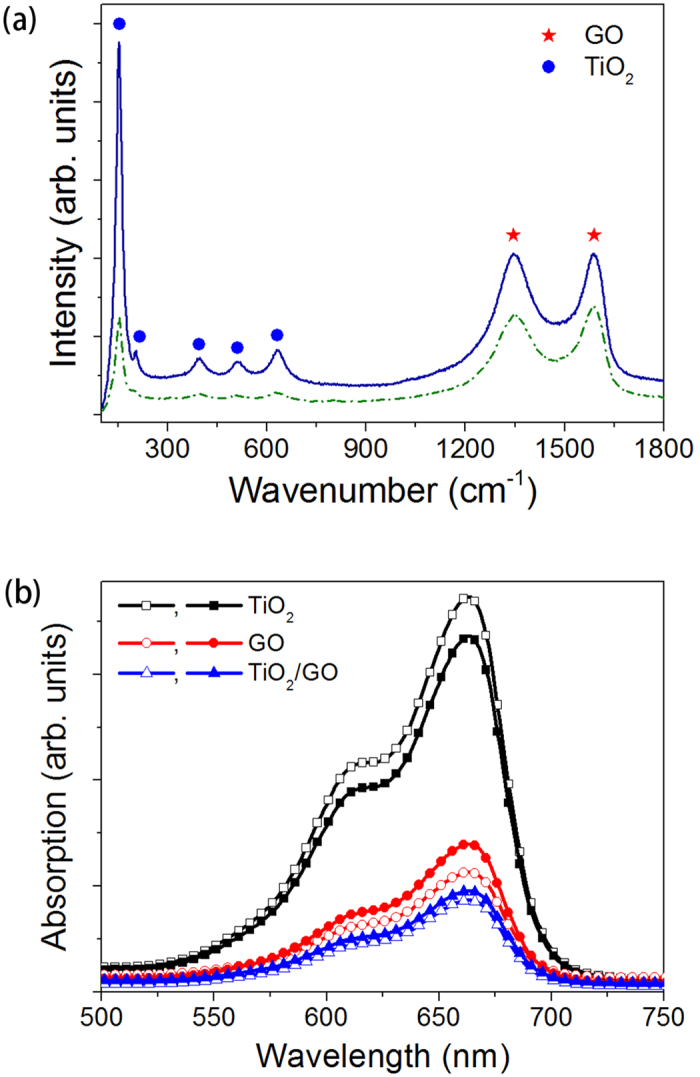
(**a**) Raman Spectra of TiO_2_/GO before (dash line) and after (solid line) photocatalysis reaction; (**b**) absorption spectra of 30 μM MB solution immersed with as-prepared (hollow symbol) and used (solid symbol) photocatalysts for 50 min.

Firstly, the TiO_2_-decorated GO sheet increased the surface area and thus MB adsorption compared to bare TiO_2_^45^,^46^. Figure 4 shows the SEM images of GO, TiO_2_ and TiO_2_-decorated GO sheet before and after photocatalytic reaction, respectively. It can be observed that all kinds of photocatalysts generally maintained the morphology during the reaction. Raman spectra of TiO_2_-decorated GO sheet shown in Fig. 5(a) indicated that the chemical bonding did not change significantly after the reaction. Absorption spectra of 30 μM MB solution immersed with the photocatalysts were also measured to characterize the surface area and MB adsorption capacity for different photocatalysts, as described in the *Methods Section* and shown in Fig. 5(b). It can be concluded from Fig. 5(b) that the MB solutions immersed with GO and TiO_2_-decorated GO sheets exhibited lower UV-Vis absorption than that immersed with bare TiO_2_, indicating a lower concentration of MB solution i.e. higher MB adsorption capacity contributed by GO. Furthermore, it should be noticed that the absorption cross section for each kind of photocatalyst did not change much after the reaction, which was consistent with SEM and Raman characterization. The consistency of SEM, Raman and UV-Vis absorption during the reaction might also imply good stability of the microreactor performance.

**Figure 6:**
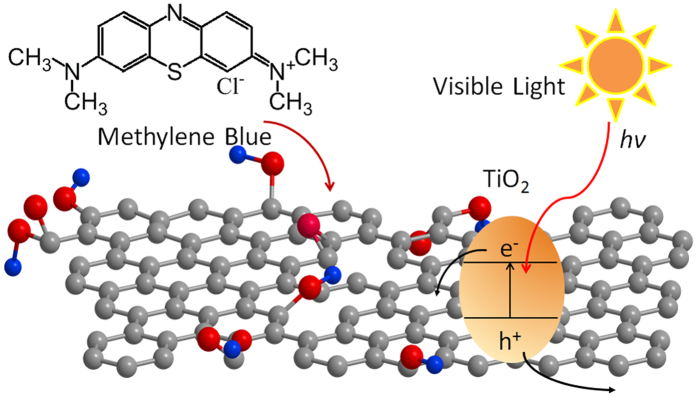
The mechanism of TiO_2_/GO photodegradation of MB.

**Figure 7:**
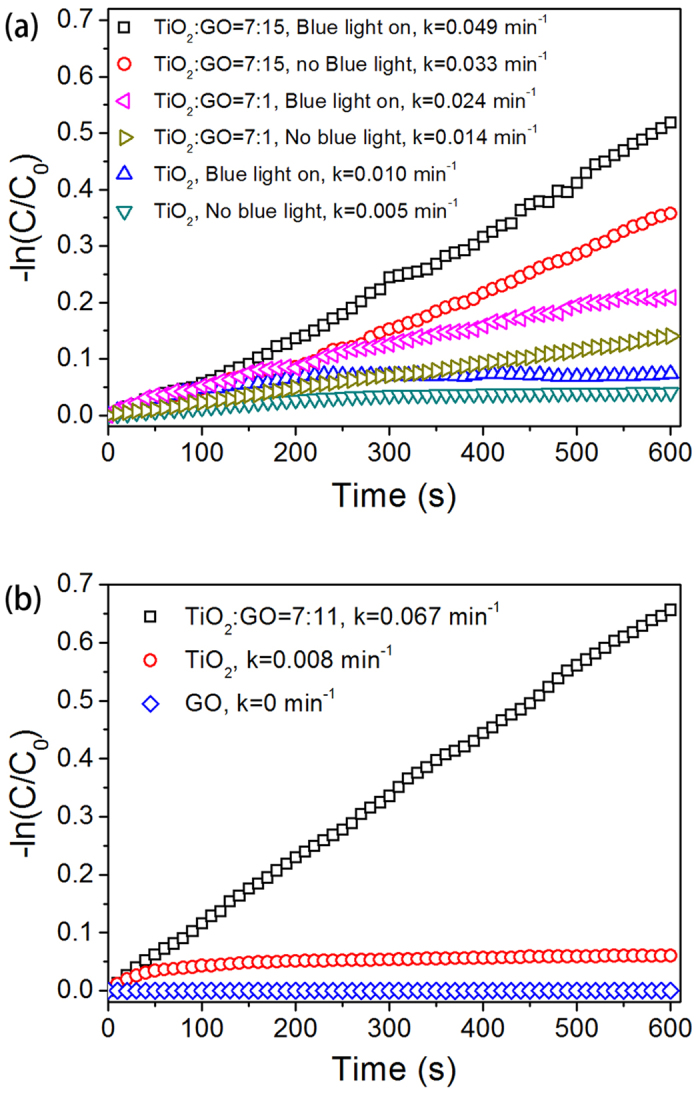
Reaction rate constant obtained from the photodegradation of MB (**a**) with and without blue LED (the photocatalytic light) switched on (**b**) after complete adsorption overnight in a 30 μM MB solution.

Secondly, the oxygen related functional groups (hydroxyl, epoxy, etc.) facilitated the binding between oxide and GO^47^, and GO could act as electron acceptors to retard the hole-electron recombination^46^,^48^, as shown in Fig. 6. To clarify the influence of each components of the whole system including the microreactor, spectroscopic light and catalytic light, the degradation of MB against reaction time with and without blue light irradiation for bare TiO_2_ and TiO_2_/GO was investigated in detail, as shown in Fig. 7(a). As a result, the adsorption of MB in the dark is enhanced by increasing GO content, indicating a higher MB adsorption capacity of the TiO_2_/GO composite. The photocatalytic activity under blue light irradiation was also found to vary with the weight mixing ratio of TiO_2_/GO.

For identifying the contribution of photocatalysis from adsorption, the photocatalytic sheets including bare TiO_2_, GO, and TiO_2_/GO with the weight mixing ratio of 7:11 were soaked overnight in a 30 μM MB solution in the dark to ensure complete adsorption. After that the photocatalytic degradation was measured. It could be observed from Fig. 7(b) that the reaction rate constant of GO was approximately zero, which confirmed that GO is not a photocatalyst for the degradation of MB. The TiO_2_/GO sheet (TiO_2_:GO = 7:11) exhibited a reaction rate constant of approximately 0.067 min^−1^, which was almost one order magnitude higher than that of bare TiO_2_ sheet (k = 0.008 min^−1^). Hence, the addition of GO into TiO_2_ enhances the photocatalytic activity rather than just improving the adsorption of MB molecules, especially with an optimized mixing ratio.

**Figure 8:**
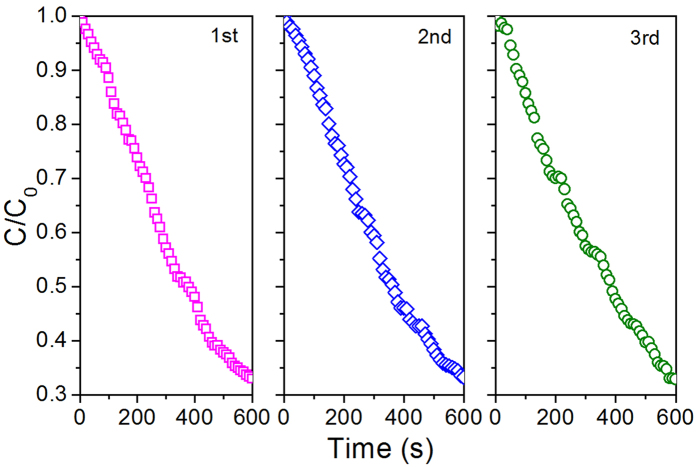
Performance reproducibility of 7:11 TiO_2_/GO microreactor.

The performance reproducibility of the microreactor was also verified. Three photodegradation cycles of MB were tested using 7:11 TiO_2_/GO microreactor. For each cycle, photocatalysis was carried out for 10 min, and the microchannel was rinsed using pure water before the next cycle. Figure 8 indicated the degradation of MB in the three cycles could exhibit acceptable reproducibility with the reaction rate constants of 0.113, 0.112, and 0.110 min^−1^, respectively, which was corresponding to SEM, Raman and UV-Vis absorption.

In conclusion, a photocatalytic microreactor was proposed for the real-time monitoring of photocatalytic activity absorption spectroscopy. The photodegradation of MB was analyzed under monochromatic photocatalytic and spectroscopic light sources, using this chip for the rapid screening of TiO_2_-decorated GO sheet catalysts. An optimized weight ratio of 7:11 in this architecture was found to exhibit the best performance. The sampling interval time was minimized to 10 s for achieving real-time detection Thus, the proposed method and apparatus are promising for the rapid screening of various photocatalysts and for the time sensitive investigation of the photocatalytic mechanism. For more details of Raman characterization and the calibration of MB concentration, please see the supplementary information file.

## Methods

### Materials

Polydimethylsiloxane (PDMS, Sylgard 184, Dow Corning Co. USA) and SU8 2075 (Microchem Co., USA.) were used to build up the architecture of the microreactor. Natural graphite powder having a size less than 10 nm was purchased from Shenzhen Sinuo Industrial Development Co., Ltd. Commercial P25 titanium dioxide powder (mesh 300, 99.9%), was purchased from Beijing Mountain Technical Co., Ltd., China. Methylene blue trihydrate was purchased from Xilong Chemical Co., Ltd., China. All reagents were analytical grade and used without further purification.

### Preparation of photocatalysts

Bare titanium dioxide (TiO_2_) and GO/TiO_2_ composite were prepared for photocatalysis. A precursor slurry containing TiO_2_ was prepared by dissolving 0.15 g of P25 titanium dioxide powder in 0.3 mL n-butanol, 0.275 mL deionized water, and 0.05 mL titanium diisopropoxide bis(acetylacetonate), followed by stirring for approximately 10 min. A moderate amount of the slurry was dropped into a trench having dimensions of 8 × 0.8 × 0.02 mm, and the overflowing slurry was removed by scraping using the edge of a glass slice. A uniform film of titanium was formed after naturally drying under air. The film was then peeled off and placed in an oven at 120 °C for 10 min to complete low-temperature sintering. The GO solution was synthesized by the modified Hummers’ method^49^: 0.5 g expanded graphite, 3 g KMnO_4_, and 0.5 g NaNO_3_ were mixed in 60 mL concentrated sulfur acid, and the solution was stirred for at least 12 h until the color changed to dark green; then, the solution was diluted using 200 mL deionized (DI) water. Next, 12 mL of H_2_O_2_ was introduced. Then, the solution was centrifuged at 10000 rpm for 5 min to remove the impurities. TiO_2_ powders and the GO solution at a concentration of 1 wt% were added to a reagent bottle with weight mixing ratios from 7:1 to 7:15. Then, the mixtures were stirred for 10 min, and the TiO_2_/GO sheets (approximately 20 μm thickness) were obtained by suction filtration.

### Device fabrication

**Figure 9:**
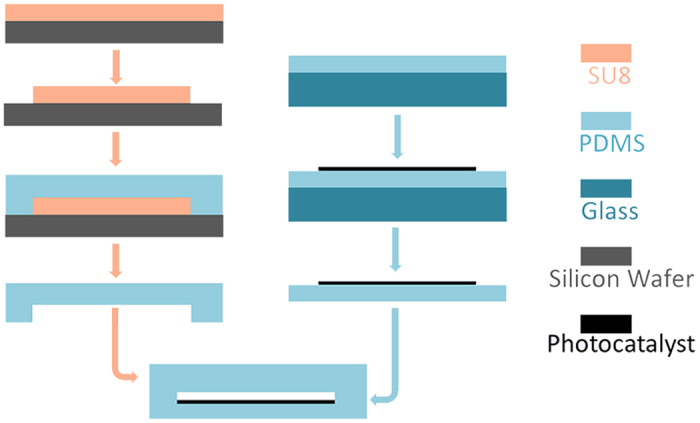
Procedure for the fabrication of the microreactor chip.

The chip was fabricated by soft lithography as shown in Fig. 9. SU-8 2075 with a thickness of approximately 250 μm was patterned on a silicon wafer to form a mold. Then, a PDMS primer was poured into the mold, and the cured PDMS film was peeled off after baking. The fluid inlet and outlet were drilled on the PDMS film. Meanwhile, a bottom PDMS film having a thickness of approximately 2 mm was prepared. The photocatalyst sheet was placed on the bottom PDMS film. Next, the top and bottom PDMS films were aligned and bonded together after oxygen plasma treatment. Both ends of the chips were cut to be smooth. Copper foils with a hole (approximately 0.8 × 0.25 mm) as the mask for probing light were fixed on both ends of the chips, while the hole on the copper foil was aligned with the fluidic channel of the chip. A device without the photocatalyst was also prepared as a control for the comparison of the photocatalysts.

### Real-time spectroscopic monitoring of the photocatalytic reaction

**Figure 10:**
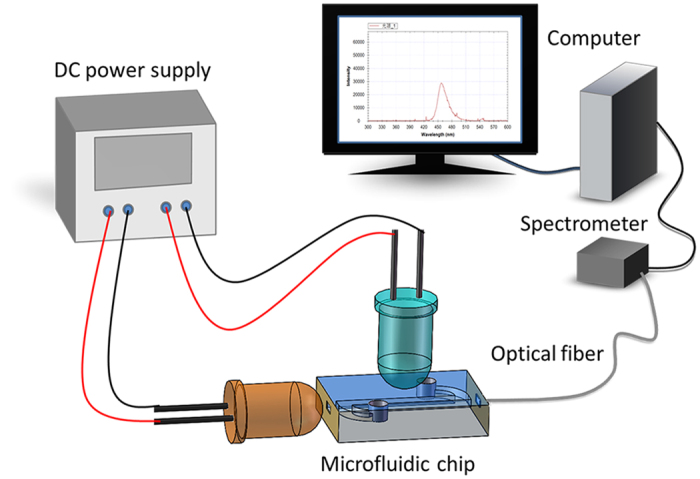
Schematic diagram of real-time photocatalytic characterization using the fabricated microreactor.

As shown in Fig. 10, the microreactor chip was placed on a three axial displacement platform. A blue LED (Φ5 mm, Epileds Co. China) with a center wavelength of 459 nm and a full-width at half-maximum (FWHM) of 25 nm was used as the photocatalytic light source, driven at DC 3.0 V for achieving a power density of approximately 28 mW/cm^2^, and placed above the microreactor. An orange LED (Φ5 mm, Epileds Co. China) with a center wavelength of 609 nm and an FWHM of 15 nm was chosen as the spectroscopic light source, driven at DC 2.0 V with a power density of approximately 24 mW/cm^2^, and aligned to the fluidic channel of the microreactor. A multimode optic fiber (200 μm) was also aligned to the fluidic channel and opposite to the spectroscopic light. The fiber was connected to a spectrometer (Spectrometer SD1200, OtO Photonics Inc., China) to measure the intensity of the spectroscopic light after it went through the channel. The increase of the measured light intensity implied the degradation of the MB solution.

At the start of the test, the microchannel in the chip was filled with DI water, and its absorption spectrum was recorded by the spectrometer at an integrated time of 50 ms. Then, DI water was pumped out, and the MB water solution at 30 μM was pumped into the microchannel by using a syringe. Next, the inlet and outlet was encapsulated by a thin PDMS film for preventing the evaporation of the solution. The blue LED was kept on for photocatalysis. The orange LED was switched on for taking absorption spectrum. Once a spectrum was taken, the orange spectroscopic LED would be switched off. In this setup, the photocatalytic activities of TiO_2_ or TiO_2_-decorated GO sheets was measured with a minimized interval at approximately 10 s.

### Surface analysis

SEM images were taken under Zeiss SUPRA 55. Raman spectra were taken under Renishaw. The surface area as well as MB adsorption capacity was characterized as follows: photocatalysts membrane fixed on Cu foil with fixed area (GO, TiO_2_ and TiO_2_/GO_7:11_), both before and after reaction, rinsed) were immersed into 30 μM MB solution for 50 min. Then UV-Vis absorption spectra of the MB solution was measured under UV-Vis spectrometer (Model UI-1800PC, MAPADA Co., China).

## Additional Information

**How to cite this article**: Li, Y. *et al*. Real-time spectroscopic monitoring of photocatalytic activity promoted by graphene in a microfluidic reactor. *Sci. Rep.* **6**, 28803; doi: 10.1038/srep28803 (2016).

## Supplementary Material

Supplementary Information
