# Supplementary material for: Real-time spectroscopic monitoring of photocatalytic activity promoted by graphene in a microfluidic reactor
Source: Sci Rep. 2016 Jun 27;6:28803. doi: 10.1038/srep28803 (PMC4921810; doi:10.1038/srep28803)
Supplement: Supplementary Information [file srep28803-s1.pdf]

Real-time spectroscopic monitoring of photocatalytic activity promoted by graphene in a microfluidic reactor

Yifan Li, Beichen Lin, Likai Ge, Hongchen Guo, Xinyi Chen, Miao Lu

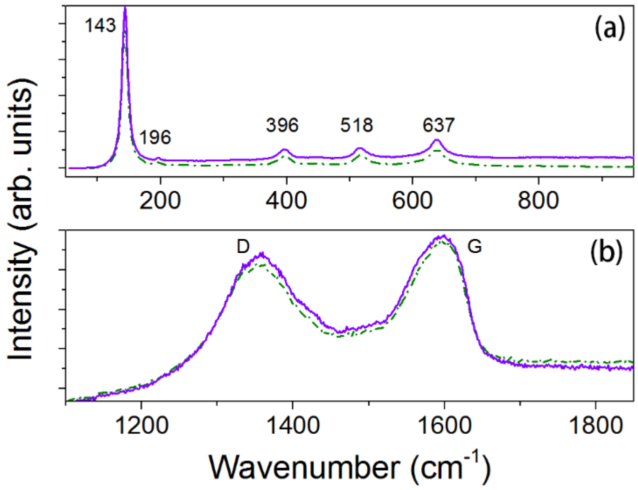

Figure S1. Raman Spectra of (a) TiO_2 and (b) GO before (dash line) and after (solid line) photocatalytic reaction

MB concentration determined by absorption spectra

In order to investigate the photocatalytic effect of the microreator, MB concentration should be quantified via absorption spectra during the whole process. According to the Beer–Lambert law, the wavelength-dependent absorbance A is described as follows:

$$A = \lg\frac{I_0}{I} = \varepsilon c L \qquad (2)$$

where I_0 and I represent the measured light intensity at the selected wavelength in the absence and presence of the sample, respectively. ε represents the wavelength-dependent molar extinction coefficient, c represents the sample concentration, and L

represents the optical path length through the sample. Hence, I_0/I is measured for the determination of the sample concentration with a known molar extinction coefficient and an optical path length.

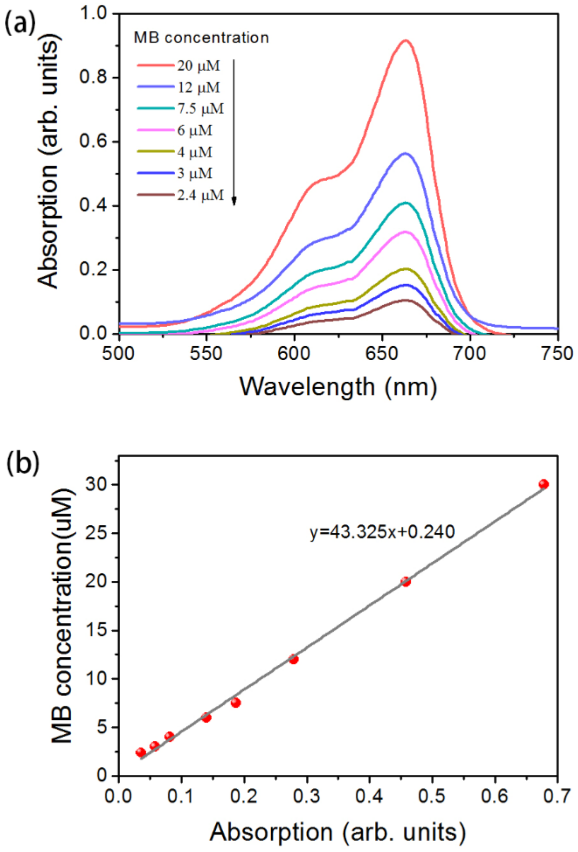

Figure S2. (a) Absorption spectra of the MB water solution at different concentrations from 2.4 μM to 30 μM, using a normal spectrometer; (b) calibrated Beer–Lambert relation of MB concentration versus absorbance at 609 nm (comparable to the spectroscopic light source in this work). The fitted slope for Beer–Lambert Law was about 43.3, which meant that a 10% drop of absorbance would be induced by a concentration decrease of approximately 4.3 μM. Thus, the MB solution concentration is determined by its absorbance at 609 nm.
